# Supplementary material for: Epigenetic Factors Related to Low Back Pain: A Systematic Review of the Current Literature
Source: Int J Mol Sci. 2023 Jan 17;24(3):1854. doi: 10.3390/ijms24031854 (PMC9915125; doi:10.3390/ijms24031854)
Supplement: Supplementary file 1 [file ijms-24-01854-s001.zip › ijms-2094058-Table S1.pdf]

Table S1. Search strategy table.

	Concept 1	Concept 2	Concept 3	Concept 4
**Key concepts**	Acute low back pain	Chronic low back pain	Neuropathic pain	Epigenetic modulation
**Free text terms / natural language terms**<br>(Synonyms, UK/US terminology, medical/laymen's terms, acronyms/abbreviations, drug brands, more narrow search terms)	Acute Lumbar spine pain<br>Acute sciatica	Chronic lumbar spine pain<br>Chronic sciatica	Radicular pain<br>Radiculopathy	Mutation<br>Epigenetic variants<br>Methylation<br>Regulation
**Subject fields**<br>(Title, abstract, keyword)	Title, abstract, keyword	Title, abstract, keyword	Title, abstract, keyword	Title, abstract, keyword
